# Evaluating the Impact of Commonly Used Pesticides on Honeybees (*Apis mellifera*) in North Gonder of Amhara Region, Ethiopia

**DOI:** 10.1155/2023/2634158

**Published:** 2023-03-30

**Authors:** Zewdie Abay, Amssalu Bezabeh, Alemayehu Gela, Asaminew Tassew

**Affiliations:** ^1^Andassa Livestock Research Center, P.O. Box 27, Bahir Dar, Ethiopia; ^2^Oromia Agricultural Research Institute, Holeta Bee Research Center, P.O. Box 22, Holeta, Ethiopia; ^3^College of Agricultural and Environmental Science, Bahir Dar University, P.O. Box 5501, Bahir Dar, Ethiopia

## Abstract

Global honeybee losses and colony decline are becoming continuous threat to the apicultural industry, as well as, for food security and environmental stability. Although the putative causes are still unclear, extensive exposure of bees to pesticides could be the possible factor for worldwide colony losses. This study was aimed at evaluating the impact of nine commonly used pesticide incidents on adult worker honeybees (*A. mellifera*) under the laboratory condition, in North Gonder of Amhara region, Ethiopia. Feeding test, contact test, and fumigation tests were carried out for each pesticide following the standard procedures, and each pesticide toxicity was compared to the standard toxic chemical, dimethoate 40% EC (positive control), and to 50% honey solution (negative control). The results revealed that all the tested pesticides caused significant deaths of the experimental bees (*P* < 0.05) in all the tests when compared to the negative control. Diazinon 60% EC, endosulfan 35% EC, and malathion 50% EC were appeared highly toxic causing 100% mortality of bees, while chlorsulfuron 75% WG killed 90% of the experimental bees as tested via feeding. On the other hand, agro-2, 4-D and its mixture with glycel 41% EC are moderately toxic, and mancozeb 80% WP and glycel 41% EC were slightly toxic to honeybees as compared to the positive control (dimethoate 40% EC). Suddenly, diazinon 60% EC and malathion 50% EC triggered 100% mortality of bees, while endosulfan 35% EC and chlorsulfuron 75% WG caused 63.63% and 90.82% of bee mortality, respectively, when evaluated via contact test. The fumigation test also showed that chlorsulfuron 75% WG, diazinon 60% EC, and endosulfan 35% EC caused 100%, 86.7%, and 65.6% mortality rate of bees. Our result also highlighted that tested LD_50_ of all pesticide incidents were significantly lower than the manufacturer-based LD_50._ This shows that local honeybees *A. m. jemenetica* are extremely sensitive to commonly used agricultural pesticides, which may affect the colony level due to the intensive application of these pesticides in Ethiopia.

## 1. Introduction

Honeybees (*Apis mellifera*) are well known for their commercial products, playing increasing roles in income generation, healthy food, and alternative medicinal values. They are not only a key contributor to economic functions but also they are the single most important species pollinator in natural ecosystems across the globe [1]. In Africa, *A. mellifera* contributes for more livelihood of the community and plays an essential role in pollinating the most of the agricultural crops [2, 3]. To this fact, about 50% of the leading global food commodities depend on pollination by honey bees for either fruit formation or seed set [4]. In this case, bees are the most efficient pollinators for most cash crops, stable food crops, vegetables, and fruit trees [5].

However, a large scale dramatic losses and decline of pollinators including honeybees have been reported in several regions of the world resulting severe threat to the apiculture industry and global food security [6–9]. For example, beekeepers in the United States lost an estimated 50.8% of their managed honey bee colonies only in 2021, which was the highest annual loss on record [10]. Although the extent is different, similar trends have been reported in African countries in recent years affecting the self-sustainability of both wild and managed bee populations [11]. Although the putative causes of colony loss are still unclear, the combined effects of climate change, intensive agriculture, pesticides use, pest and pathogens, and biodiversity loss are some risk factors for global honeybee loss [12]. Earlier reports suggested that the extensive exposure of bees to pesticide incidents would possibly be a major factor for honeybee loss and colony decline [13–17]. In Ethiopia, widespread reports indicate that exposure to commonly used agricultural pesticides has been linked to the dramatic honeybee deaths and colony decline than any other factors in the country [18–21]. Such losses of honeybees have in turn resulted in reduction of honey production as well as crop production, through disrupting pollination services [22].

In general, both managed and wild honeybees are exposed to a wide range of pesticide incidents, which can only be determined through extensive toxicological assessments [23]. In previous studies, a number of pesticide incidents were investigated in several countries of the world [9, 24, 25]. The majority of investigations showed that honeybees frequently became exposed to chemical pesticides as a result of their foraging activity. However, some studies suggest that there are three key pathways of poisoning incidents. The primary incident occurs when forager bees come into direct contact with pesticides that are applied to plants, and the bees rapidly die in the field [26, 27]. A second possible route of pesticide incident happens when forager bees bring contaminated nectar, pollen, and water sources into the hives; thus, the entire colony can be affected by the contaminated material [28, 29]. The third possible exposure of pesticides happens via aerial spray drift [27]. As a result, the measurement of toxic effects of most pesticides has relied largely on the determination of acute toxicity than chronic and sublethal effects due to its rapid appearance of visible symptoms [30]. Acute pesticide tests via ingestion (feeding), contact exposure, or ambient air drifting intake are, therefore, common tests for pesticide incidents [31, 32]. Contact exposure and ingestion are well studied routes of contamination that reveal pesticide-specific effects on honeybee health [33, 34]. Nevertheless, exposure of bees to pesticide through air drifting (fumigation test) is thought to be a minor route of pesticide uptake due to volatile nature of some pesticide components [35].

In Ethiopia, intensive application of commonly used agricultural pesticides against pests and weeds control has been largely reported [36–38]. Such open field application of agricultural pesticide incidents has been suspected for most exposure and a flagship poisoning of honeybees in the country. The majority of farmers in Ethiopia follow an indiscriminate application of pesticides over the open agricultural fields, even during the visiting period of forager bees on the same field. As a result, beekeepers have been continuously reporting the deaths of honeybees and colony population declining. However, the acute toxicity tests and agricultural pesticide incidents at certain concentration level are not yet studied in Ethiopia.

Therefore, this study was aimed to determine the acute toxicity of nine commonly used pesticide incidents on local honeybees (*Apis mellifera jemenitica*) under laboratory condition in Chilga district of Northern Gonder, Amhara region, Ethiopia. Understanding the pesticide poisoning incidents through different mode of exposures can help to design and implement best management practices in the potential sources of exposure areas.

## 2. Materials and Methods

### 2.1. Pesticide Selection

Pesticide selection was carried out based on their distribution, wide application, target use, and their market channel in Chilga district of Northern Gonder, Amhara regional state, Ethiopia. Accordingly, nine commonly used pesticides including agro-2,4-D, glycel 41%, diazinon 60% EC, chlorsulfuron 75% WG (or slean 75% WG), mixture of agro-2, 4-D and glycel 41% EC, mancozeb 80% WP, malathion 50% EC, endosulfan 35% EC, and dimethoate 40% EC were purchased from local markets as well as from veterinary drug stores of the Chilga district, Norther Gonder, Amhara region. The collected pesticides were transported to the regional animal health laboratory and stored at the room temperature (25°C) until the acute toxicity tests were performed.

### 2.2. Bee Samples

Adult worker honeybees were collected at early in the morning from strong and preassumed healthy colonies based on their activity and internal inspection. The sampled bees were taken to laboratory using well ventilated plastic jars. The bees starved for about 2 hrs prior to the commencement of the laboratory experiment in order to induce their pesticide contaminated solution consumption rate.

### 2.3. Laboratory Test

**Figure 1:**
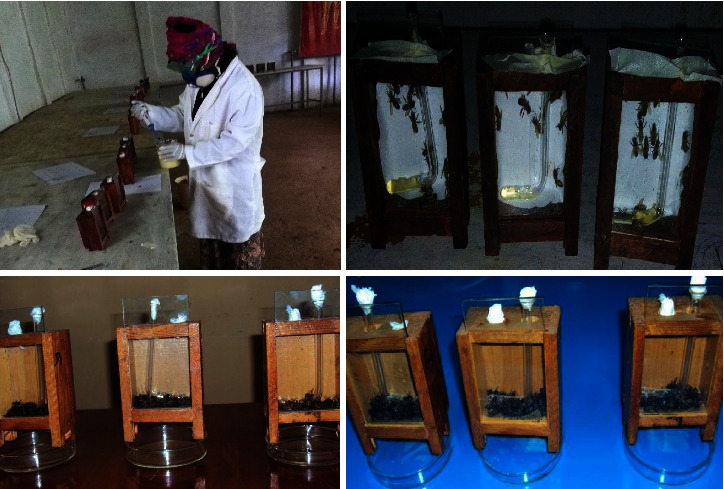
Laboratory tests and response of honeybees (*A. mellifera*) to commonly used agricultural pesticides.

Acute toxicity of selected nine pesticides (eight widely used and one standard toxic pesticide used as the control) was tested in the laboratory on local honeybees (*Apis mellifera jemenitica*) via feeding, contact, and vapor tests following the standard laboratory procedure [39, 40] (Figure 1). For this purpose, the collected adult worker bees were anesthetized with Co_2_ and inserted to well ventilated laboratory cages (size 5.5 × 8.5 × 10 cm), and placed at room temperature (25 ± 2°C) and humidity (60–70%) during study periods. The mortalities caused by individual pesticides were compared with the positive control (dimethoate 40% EC), negative control (water), and amongst the test pesticides using the following mode of tests.

#### 2.3.1. Feeding Test

To determine the toxicity effect of each chemical via feeding test, 30 predetermined healthy worker bees were placed in laboratory cages. Then, the bees were provided with 50% honey solution containing the recommended concentration of 300 *μ*g (logically estimated as 10 *μ*g/bee) of each test pesticide to determine the toxicity effect according to the procedure of Medrzycki et al. [41]. The recommended concentration of each test pesticide is indicated in Table 1, and each treatment was replicated 3 times. Both the number of dead and injured bees were recorded after 15, 30, and 45 minutes, then after 1, 2, 4, 6, 12, 24, and 48 hrs, and compared with negative control (50% honey solution) and positive standard toxic chemicals (dimethoate 40% EC). Honey solution was replenished for all experimental bees in all test categories whenever required (when they finished the supplied resource) [42].

#### 2.3.2. Contact Test

In the mode of contact test, filter papers were immersed in each recommended concentration (Table 1) of test pesticides and allowed to be air dried at room temperature. The filter papers containing test pesticides were enclosed separately in the lab cage containing 30 worker bees. Toxicity effects of each concentration of test materials were then compared with 0.3% standard chemicals and the control (paper immersed in pure water). Each treatment was replicated three times as described by Gough et al. [42]. Then, every activity of bees after the application of each test was observed to determine the physiological and behavioral effects of pesticides on experimental bees.

#### 2.3.3. Vapor or Fumigation Test

For fumigation test, another 30 worker bees were held in laboratory cage and placed over the Petri dish filled with recommended concentration (Table 1) of each pesticide with three replications. The number of dead and injured bees was recorded in an hour interval for two days. Then, the death rate of bees was compared with the standard toxic chemical (dimethoate 40% EC) known to kill 100% of bees at concentration level of 0.3% and with nontoxic control (Petri dish filled with water). Similar to feeding test, all the experimental bees in the cages were fed 50% natural honey solution throughout the entire experimental period [42].

Finally, percent of mortality rates caused by each pesticide in each mode of test was corrected by Abbott formula [43] as indicated as follows:

(1) $$\begin{array}{c} \%\;of\;mortality:Correct\;mortality\;\left(Abbott\right)=\frac{\%\;mortality\;treatment-\%\;mortality\;control\;x100}{100-Mortality\;control}\text{.} \end{array}$$

#### 2.3.4. Data Management and Statistical Analysis

The variances of laboratory data analyzed using GLM and Tukey's honest significant difference (HSD) at 5% level of significance were used for mean separation whenever significant results were encountered.

## 3. Results and Discussions

### 3.1. Behavioral and Physiological Effect of Pesticides

In this study, exposure to each pesticide incident appears to impair the behavioral and physiological response of experimental bees in the cages immediately after the exposure (Figure 1). We observed that all the experimental bees suffered seriously except for those tested with water control. Typical symptoms for bees suffered due to pesticide exposure include high disturbance, narcotization, hovering sound, and crawling in the bottom wall of the test cages. This result highly agrees with the findings of Thompson [44], who reviewed a wide behavioral effects and potential risks of pesticide incidents on bees following their exposure. Experimental bees in the feeding test were showed high disturbance and narcotization than those in contact and fumigation tests. Fortunately, the bees showed no trophallactic transfer of food from each other as soon as they recognized contamination in the food solution either to save themselves or loss their cognitive behavior [45, 46]. Evidence from recent study also highlighted those pesticide-induced cognitive impairments on olfactory learning, visual learning, and memory of honeybees [7]. Similarly, acute exposure of bees to neonicotinoid induces a series of symptoms that are consistent with hyper-responsive neural impairments [47]. In this case, experimental bees exposed to all the test chemicals showed reduced proboscis extension as compared to the unexposed bees.

Apart from behavioral responses, bees exposed to pesticides showed some observable physiological disruptions. Almasri et al. [48] explained that even mild exposure to pesticides can directly alter the physiological homeostasis of bees and particularly if the individuals exhibit a lack the core microbiota. Meanwhile, such behavioral alteration and physiological disruption caused due to pesticide exposure directly lead to lethal effects on bees at varying time intervals for different ages of bees [13, 14, 48].

### 3.2. Acute Toxicity of Pesticides

In this study, significant acute toxicity of pesticides was recorded causing the experimental honeybees' mortality rate in all the three modes of tests (Table 2). However, there was significant toxicity difference (*P* < 0.001) among all the tested chemicals in causing the mortality of bees within the given time intervals. Diazinon 60% EC caused 37.8% and 58.9% at 15 min and 30 min experimental time, respectively, and glycel 41% EC + 2,4-D caused 10% mortality after 2 hrs of exposure to pesticides, which is significantly different in modes of action and duration (Table 2). This implies that the mode of chemical application and exposure time of bees to the pesticide incidents were differently affecting the bee's lifespan [49]. We also observed that, bees are more significantly susceptible to poisoning incidents of the pesticides when ingested the pesticides than via fumigation or body contact tests. In general, the honeybees are exposed to pesticide incidents either through direct contact with pesticides applied to plants during pollen and nectar collection in the field [26], or through food contamination with the incoming pollen or nectar in the hive [29]. The significant mortality of experimental bees observed in our current investigation could be an indicator for these routs of incident poisoning that has been causing colony losses and decline in Ethiopia.

#### 3.2.1. Feeding Test

During the feeding test, diazinon 60% EC, endosulfan 35% EC, and malathion 50% EC caused highly poisoning incidents and killed about 100% of experimental bees within shorter test periods (which is in less than an hour), while chlorsulfuron 75% WG killed about 90% of the experimental honeybees (Figure 1). Thus, these pesticide incidents were comparable to highly toxic standard pesticide (dimethoate 40% EC), but negatively act when compared to the water control. This implies that pesticides used by the farmers were fast acting and killed honeybees even before the mid-day if applied in the morning. These findings were partially agreed with the previous findings of Bezabeh and Gela [50] that stated endosulfan 35% EC and diazinon 60% EC are highly toxic incidents to honeybees of the central highlands during the same laboratory test.

**Figure 2:**
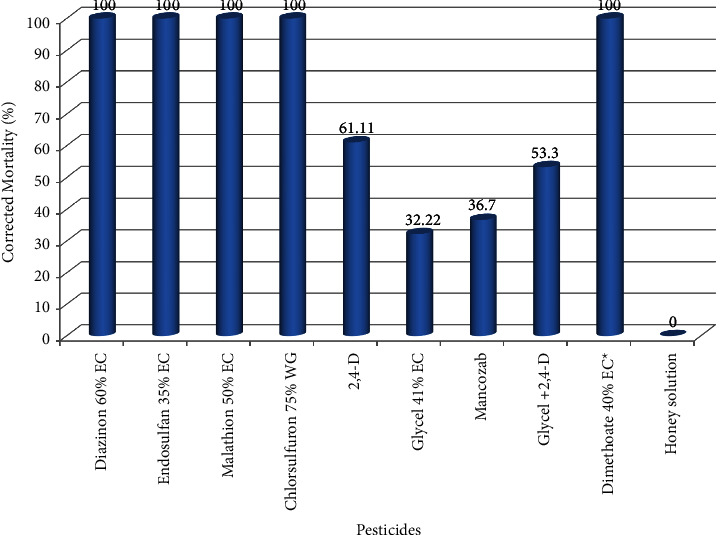
Mortality of local bees (*A. m. jemenitica*) tested via feeding.

Herbicides 2,4-D and glycel 41% EC + 2,4-D killed more than 50% of the experimental honeybees, while fungicide mancozeb 80% WP, and herbicide glycel 41% EC killed 36.7% and 32.22% of the experimental bees, respectively, when ingested with sugar solution. These results indicate that 2, 4-D, and glycel 41% EC + 2,4-D, mancozeb 80% WP, and glycel 41% EC caused significantly poisoning incidents to the local honeybees as compared to the negative control (*P* < 0.05). The mixture of glycel and 2,4-D is highly significantly more toxic to honeybees than glycel (*P* < 0.0001) and is comparable to 2,4-D (*P*=0.991) (Figure 2 and Table 3). In the previous study, 2,4-D was reported as nontoxic pesticide among the central highland bees, *A. m. bandasii* [50], but it was found to be toxic to *A*. *m. jemenitica*, while the dose formulation and application method were in the same procedure. These poisoning differences might be due to differences in geographical races of the bees adapting to specific stressors. In general, this experimental test suggests that agricultural pesticides might cause severe honeybee deaths during their application period unless necessary precautions are taken.

#### 3.2.2. Contact Toxicity Test

**Figure 3:**
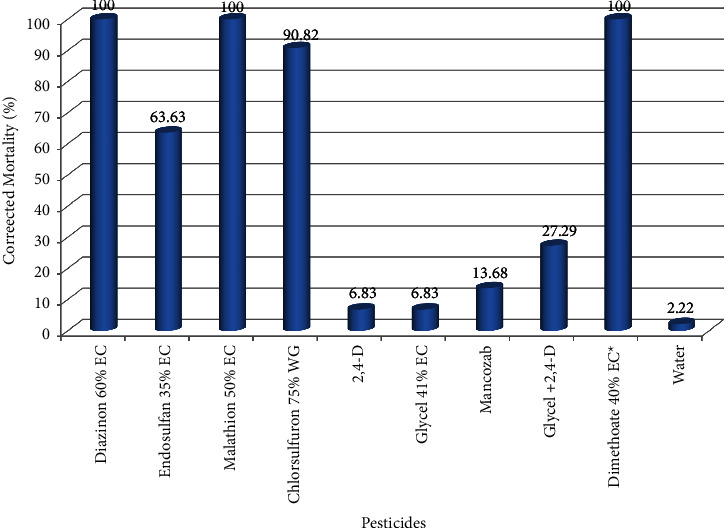
Mortality of local bees (*A. m. jemenitica*) tested via contact.

Contact toxicity analyses of the same nine pesticide incidents listed above were evaluated against the standard highly toxic pesticide (dimethoate 40% EC) and the negative control (nontoxic, water). Laboratory contact toxicity results revealed that there is highly significant difference between the negative control and pesticide incidents and among each pesticide (*P* < 0.001). Diazinon 60% EC and malathion 50% EC caused 100% mortality via contact (Figure 3), while endosulfan 35% EC and chlorsulfuron 75% WG killed 63.63% and 90.82% experimental bees, respectively, (Figure 3) and except endosulfan 35% EC, all were comparably highly toxic like that of standard insecticides, dimethoate 40% EC (*P*=0.829 − 1.00). This is in agreement with the findings of Melisie et al. [51] which showed that diazinon 60% EC and malathion 50% EC were highly toxic to honeybees when tested via contact. On the other hand, there was no significant contact toxicity difference between the negative control, water, and 2,4-D, glycel 41% EC, and mancozeb 80% WP through contact test (Table 4).

#### 3.2.3. Vapor/Fumigation Test

**Figure 4:**
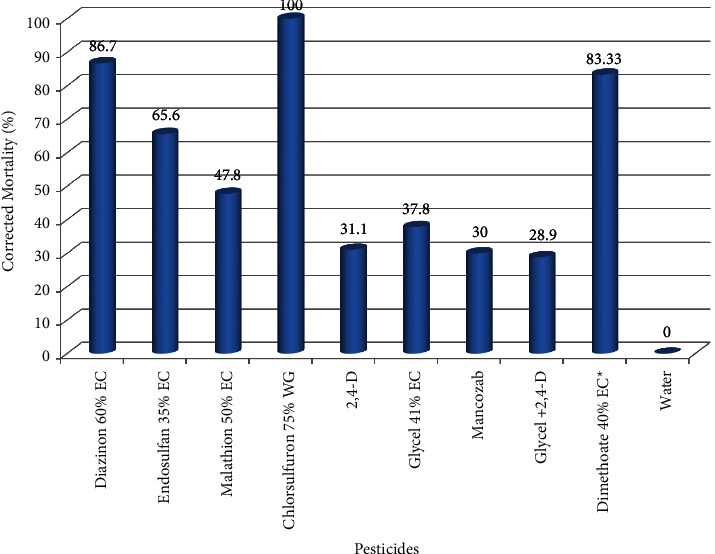
Mortality of local honeybees (*A. m. jemenitica*) when tested via fumigation.

Laboratory test of pesticides via vapor or fumigation revealed that all pesticide incidents caused significant mortality (*P* < 0.002) on local honeybees (*A. m. jemenitica*) as compared to the control treatments (water). Particularly, mortality caused due to chlorsulfuron 75% WG (100%), diazinon 60% EC (86.7%), and endosulfan 35% EC (65.6%) was significantly greater than all pesticide incidents tested and was comparable to toxic standards (dimethoate 40% EC) (Figure 4 and Table 5). This indicates that these pesticide incidents cause substantial honeybee mortality through vapor, which may be attributed to their fumy properties. This result is partiality in agreement with the work of Melisie et al. [51], who indicated that some chemicals including diazinon 60% EC have potential to volatize even at room temperature, and Bezabeh and Gela [50], who showed that diazinon 60% EC caused high mortality on the central highlands honeybees, *A, m. bandasii* through vapor and ingestion. On the other hand 2,4-D, glycel 41% EC, 2,4-D + glycel 41% EC, mancozeb 80% WP, and malathion 50% EC were less poisonous pesticides than that of toxic standard and they are moderate toxic to honeybees via vapor.

### 3.3. LD_50_ for Feeding Test

In this study, LD_50_ of each pesticide was evaluated to support the findings of the acute toxicity test on experimental bees. The LD_50_ of diazinon 60% EC, endosulfan 35% EC, malathion 50% EC, and chlorsulfuron 75% WG was less than 0.1 *μ*l/bee (Table 6) indicating that these pesticide incidents were in the standard range of highly toxic substances (LD_50_ < 2 *μ*l/bee) [52]. However, the manufacturer-based LD_50_ of these pesticides varies as follows: 0.38 *μ*g/bee for malathion 50% EC, 1.44 mg/kg/duck for diazinon 60% EC, 31–243 mg/kg/bird, and 30 mg/kg/rat for endosulfan 35% EC (Table 1). This shows that commonly used pesticide incidents in Ethiopia were highly poisoning at less concentration than recommended doses and classified as high toxic pesticides on bees than other animals as recommended by manufacturers. As a result, forager bees are more vulnerable to these poisonous pesticides as compared to other colony members because of their foraging behavior at hotspot areas of pesticide applications. It is expected that some of the foragers may not even return back to hive due to rapid action of these pesticide incidents and thereby causing colony reduction and sever loss of foragers due to these pesticides. In contrast, the LD_50_ of 2,4-D, and glycel 41% EC was tested between 6–8 *μ*l/bee and 5–7 *μ*l/bee, respectively, but manufacturer-based LD_50_ for both pesticides is 100 *μ*g/bee (Table 1). This also indicates the sensitivity of local honeybees to these pesticides at very less concentration than recommended LD_50_ value of manufacturers. While LD_50_ of mancozeb 80% WP was ranged between 33–44 *μ*l/bee, which is less than manufacturer LD_50_ 85.3 *μ*g/bee (Table 1) and, hence, classified as slightly toxic pesticides (Table 6). In Ethiopia, farmers have used the mixture of 2,4-D, and glycel 41% EC + 2,4-D against herbicides, but caused mild effects on honeybees. Atkins et al. [53] suggested that this mixture can also be used in the vicinity of bees if dosage, timing, and method of application are in accordance with instructions, but should not be applied directly on bees in the field or on colonies.

## 4. Conclusion

Generally, all the evaluated pesticides which are widely used in the study area (Chilga district) were toxic to local honeybees (*A. m. jemenitica*) with different toxicity levels. Diazinon 60% EC, endosulfan 35% EC, malathion 50% EC, and chlorsulfuron 75% WG poisoning incidents were fast acting and highly toxic to honeybees when tested via feeding and contact. Except malathion 50% EC incident that caused relatively slight toxic effect on honeybees, all other tested pesticide incidents remained highly toxic to honeybees through vapor test. Moreover, 2,4-D and mixture of glycel 41% EC plus 2,4-D are moderately toxic, while mancozeb 80% WP and glycel 41% EC are slightly poisoning incidents to the local honeybees via feeding. Chlorsulfuron 75% WG is herbicide that has been imported illegally to the country and found to be highly toxic to honeybees of the area through all exposure means (feeding, contact, and vapor). In general, this study demonstrated that the tested LD_50_ of all pesticide incidents was significantly lower than the manufacturer-based LD_50_ suggesting that local honeybees *A. m. jemenitica* are highly sensitive to the commonly used agricultural pesticides in Ethiopia.

Therefore, it is an urgent condition to enforce the existing policies, to control, and regulate illegal pesticide marketing, developing policy to exclude the misuse of highly toxic pesticide incidents and set stringent criteria for registration and marketing of less harmful products. As a result, extreme application, illegal import, sale, and distribution of those toxic pesticides should be under strict regulation law enforcement.

## Figures and Tables

**Table 1:** Description of tested pesticides as calculated from prescribed dilution rate of each pesticide. LD_50_ indicates the dose of formulated pesticides per unit body weight of an animal and is expressed as milligrams per kilogram (mg/kg) (source: https://wsdot.wa.gov/sites/default/files/2021-10/Herbicides-factsheet-Chlorsulfuron).

Trade name	Common name	Manufacturers of each pesticide	Recommended concentration	Manufacturer-based oral LD_50_	Pesticide category
Diazol 60% EC	Diazinon60% EC	Adama Makhteshim Ltd, Israel	0.5 ml/50 mlH_2_O	1.44 mg/kg for mallard duck	Insecticide
Thionex 35% EC	Endosulfan 35% EC	Seo Han chemical Co. Ltd., Seoul, Korea	0.5 ml/50 mlH_2_O	31–243 mg/kg for bird spp. 30 mg/kg rat	Insecticide
Malathion 50% EC	Malathion	Cheminova AS, Denmark	0.5 ml/50mlH_2_O	0.38 *μ*g/bee	Insecticide
Chlorsulfuron^*∗*^ 75% WG	Slean 75% WG	Sino agrochemical industry ltd, China	0.1 gm/2500 mlH_2_O	>2,000 mg/kg for rat	Herbicide
2,4-D amine 720 g/l A.E	2,4-D 720 g/l AE	Ajn agrochoice Co. Ltd-Tanzania	0.5 ml/80 mlH_2_O	>100 *μ*g/bee	Herbicide
Glycel 41% EC	Glyphosate 360 G/L SL	Excel industries limited India	0.5 ml/31.25 mlH_2_O	>100 *μ*g/bee	Herbicide
Unizeb 80% WP	Mancozeb 80% WP	Unifarma (Bangladesh) industries	1 gm/500 mlH_2_O	85.3 *μ*g/bee	Fungicide
Agrothoate 40% EC^*∗∗*^	Dimethoate 40% EC	Asiatic agricultural industries, Singapore	0.125 ml/37.5 mlH_2_O	0.10–0.35 *μ*g/bee	Insecticide
Glycel 41% EC + 2,4-D amine 720 g/l^*∗∗∗*^	Mixture of glycel 41% and 2,4-D amine 720 g/l	Local mixture	0.5 ml (0.25 ml glycel and 0.25 ml 2,4-D)/100 mlH_2_O	N/A	Herbicide

*Note.* ^*∗*^:illegally introduced herbicide; ^*∗∗*^: standard toxic chemicals used as control; ^*∗∗∗*^: mixture used by local farmers.

**Table 2:** Cumulative mortality test of bees in a given time intervals during feeding test.

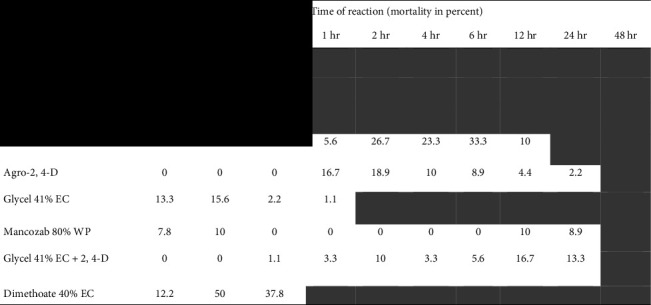

*Note.* 0 = indicates no dead bees and highlighted empty spaces indicate time intervals after 100% bee mortality.

**Table 3:** Feeding test (ingestion) multiple comparisons using Tukey's HSD.

Types of pesticides	2,4-D	Dimethoate 40% EC	Chlorsulfuron 75% WG	Diazinon 60% EC	Glycel + agro-2,4-D	Glycel 41% EC	Malathion 50% EC	Mancozeb 80% WP	Endosulfan 35% EC	Control (water)
2,4-D		−38.9000^*∗*^	−38.9000^*∗*^	−38.900^*∗*^	7.7667	28.8667^*∗*^	−38.9000^*∗*^	24.4333	−38.9000^*∗*^	61.1000^*∗*^
		*P*=0.003	*P*=0.003	*P*=0.003	*P*=0.991	*P*=0.042	*P*=0.003	*P*=0.125	*P*=0.003	*P*=00.000
Dimethoate 40% EC	38.9000^*∗*^		00.0000	00.0000	46.6667^*∗*^	67.7667^*∗*^	00.0000	63.3333^*∗*^	00.0000	1000.0000^*∗*^
	*P*=0.003		*P*=10.000	*P*=10.000	*P*=00.000	*P*=0.000	*P*=10.000	*P*=00.000	*P*=10.000	*P*=00.000
Chlorsulfuron 75% WG	38.9000^*∗*^	0.0000		0.0000	46.6667^*∗*^	67.7667^*∗*^	0.0000	63.3333^*∗*^	0.0000	1000.0000^*∗*^
	*P*=0.003	*P*=10.000		*P*=10.000	*P*=0.000	*P*=0.000	*P*=10.000	*P*=0.000	*P*=10.000	*P*=0.000
Diazinon 60% EC	38.9000^*∗*^	0.0000	0.0000		46.6667^*∗*^	67.7667^*∗*^	0.0000	63.3333^*∗*^	0.0000	53.3333^*∗*^
	*P*=0.003	*P*=10.000	*P*=10.000		*P*=0.000	*P*=0.000	*P*=10.000	*P*=0.000	*P*=10.000	*P*=0.000
Glycel + agro-2,4-D	−7.7667	−46.6667^*∗*^	−46.6667^*∗*^	−46.6667^*∗*^		21.1000	−46.6667^*∗*^	16.6667	−46.6667^*∗*^	53.3333^*∗*^
	*P*=0.991	*P*=0.000	*P*=0.000	*P*=0.000		*P*=0.257	*P*=0.000	*P*=0.551	*P*=0.000	0.000
Glycel 41% EC	−28.8667^*∗*^	−67.7667^*∗*^	−67.7667^*∗*^	−67.7667^*∗*^	−21.1000		−67.7667^*∗*^	−4.4333	−67.7667^*∗*^	32.2333^*∗*^
	*P*=0.042	*P*=0.000	*P*=0.000	*P*=0.000	*P*=0.257		*P*=0.000	*P*=10.000	*P*=0.000	*P*=0.017
Malathion 50% EC	38.9000^*∗*^	0.0000	0.0000	0.0000	46.6667^*∗*^	67.7667^*∗*^		63.3333^*∗*^	0.0000	1000.0000^*∗*^
	*P*=0.003	*P*=10.000	*P*=10.000	*P*=10.000	*P*=0.000	*P*=0.000		*P*=0.000	*P*=10.000	*P*=0.000
Mancozeb 80%	−24.4333	−63.3333^*∗*^	−63.3333^*∗*^	−63.3333^*∗*^	−16.6667	4.4333	−63.3333^*∗*^		−63.3333^*∗*^	36.6667^*∗*^
	*P*=0.125	*P*=0.000	*P*=0.000	*P*=0.000	*P*=0.551	*P*=10.00	*P*=0.000		*P*=0.000	*P*=0.005
Endosulfan 35% EC	38.9000^*∗*^	0.0000	0.0000	0.0000	46.6667^*∗*^	67.7667^*∗*^	0.0000	63.3333^*∗*^		1000.0000^*∗*^
	*P*=0.003	*P*=10.000	*P*=10.000	*P*=10.000	*P*=0.000	*P*=0.000	*P*=10.000	*P*=0.000		*P*=0.000
Control (water)	−61.1000^*∗*^	−1000.000^*∗*^	−1000.000^*∗*^	−1000.000^*∗*^	−53.3333^*∗*^	−32.233^*∗*^	−1000.000^*∗*^	−36.667	−1000.000	
	*P*=0.000	*P*=0.000	*P*=0.000	*P*=0.000	*P*=0.000	*P*=0.017	*P*=0.000	*P*=0.005	*P*=0.000	

**Table 4:** Contact test multiple comparisons using Tukey's HSD.

Types of pesticides	2,4-D	Dimethoate 40% EC	Chlorsulfuron 75% WG	Diazinon 60% EC	Glycel + 2,4-D	Glycel 41% EC	Malathion 50% EC	Mancozeb 80% WP	Endosulfan 35% EC	Control (water)
2,4-D		−91.1000^*∗*^	−82.2000^*∗*^	−91.1000^*∗*^	−200.0000^*∗*^	0.000	−91.1000^*∗*^	0.0000	−55.5333^*∗*^	6.6667
		*P*=0.000	*P*=0.000	*P*=0.000	*P*=0.043	*P*=10.000	*P*=0.000	*P*=10.000	*P*=0.000	*P*=0.963
Dimethoate 40% EC	91.1000^*∗*^		8.9000	0.0000	71.1000^*∗*^	91.1000^*∗*^	0.0000	91.1000^*∗*^	35.5667	97.7667^*∗*^
	*P*=0.000		*P*=0.829	*P*=10.000	*P*=0.000	*P*=0.000	*P*=10.000	*P*=0.000	*P*=0.000	*P*=0.000
Chlorsulfuron 75% WG	82.2000^*∗*^	−8.9000		−8.9000	62.2000^*∗*^	82.2000^*∗*^	−8.9000	82.2000^*∗*^	26.6667^*∗*^	88.8667^*∗*^
	*P*=0.000	*P*=0.829		*P*=0.829	*P*=0.000	*P*=0.000	*P*=0.829	*P*=0.000	*P*=0.003	*P*=0.000
Diazinon 60% EC	91.1000^*∗*^	0.0000	8.9000		71.1000^*∗*^	91.1000^*∗*^	0.0000	91.1000^*∗*^	35.5667^*∗*^	97.7667^*∗*^
	*P*=0.000	*P*=10.000	*P*=0.829		*P*=0.000	*P*=0.000	*P*=10.000	*P*=0.000	*P*=0.000	*P*=0.000
Glycel + 2,4-D	200.000	−71.1000^*∗*^	−62.2000^*∗*^	−71.1000^*∗*^		200.000	−71.1000^*∗*^	^*∗*^200.00	−35.5333^*∗*^	26.6667^*∗*^
	*P*=0.043	*P*=0.000	*P*=0.000	*P*=0.000		*P*=0.043	*P*=0.000	*P*=0.043	*P*=0.000	*P*=0.003
Glycel 41% EC	0.0000	−91.1000^*∗*^	−82.2000^*∗*^	−91.1000^*∗*^	−200.00		−91.1000^*∗*^	0.0000	−55.5333^*∗*^	6.6667
	*P*=10.00	*P*=0.000	*P*=0.000	*P*=0.000	*P*=0.043		*P*=0.000	*P*=10.000	*P*=0.000	*P*=0.963
Malathion 50% EC	91.1000^*∗*^	0.0000	8.9000	0.0000	71.1000^*∗*^	91.1000^*∗*^		91.1000^*∗*^	35.5667^*∗*^	97.7667^*∗*^
	*P*=0.000	*P*=10.000	*P*=0.829	*P*=10.000	*P*=0.000	*P*=0.000		*P*=0.000	*P*=0.000	*P*=0.000
Mancozeb 80% WP	0.0000	−91.1000^*∗*^	−82.2000^*∗*^	−91.1000^*∗*^	−200.0	0.0000	−91.1000^*∗*^		−55.5333^*∗*^	6.6667
	*P*=10.00	*P*=0.000	*P*=0.000	*P*=0.000	*P*=0.043	*P*=10.00	*P*=0.000		*P*=0.000	*P*=0.963
Endosulfan 35% EC	55.5333^*∗*^	−35.5667^*∗*^	−26.6667^*∗*^	−35.5667^*∗*^	35.5333^*∗*^	55.5333^*∗*^	−35.5667^*∗*^	55.5333^*∗*^		62.2000^*∗*^
	*P*=0.000	*P*=0.000	*P*=0.003	*P*=0.000	*P*=0.000	*P*=0.000	*P*=0.000	*P*=0.000		*P*=0.000
Control (water)	−6.6667	−97.7667^*∗*^	−88.8667^*∗*^	−97.7667^*∗*^	−26.6667^*∗*^	−6.6667	−97.7667^*∗*^	−6.6667	−62.2000^*∗*^	
	*P*=0.963	*P*=0.000	*P*=0.000	*P*=0.000	*P*=0.003	*P*=0.963	*P*=0.000	*P*=0.963	*P*=0.000	

**Table 5:** Vapor (fumigation) multiple comparisons using Tukey's HSD.

Types of pesticides	2,4-D	Dimethoate 40% EC	Chlorsulfuron 75% WG	Diazinon 60% EC	Glycel + 2,4-D	Glycel 41% EC	Malathion 50% EC	Mancozeb 80% WP	Endosulfan 35% EC	Control (water)
2,4-D		−52.2100^*∗*^	−68.8667^*∗*^	−55.5333^*∗*^	2.2333	−6.6333	−16.6667	1.1333	−34.4000^*∗*^	31.1333^*∗*^
		*P*=0.000	*P*=0.000	*P*=0.000	*P*=10.00	*P*=0.974	*P*=0.151	*P*=10.000	*P*=0.000	*P*=0.001
Dimethoate 40% EC	52.2100^*∗*^		−16.6567	−3.3233	54.4433^*∗*^	45.5767	35.5433^*∗*^	53.3433^*∗*^	17.810	83.3433^*∗*^
	*P*=0.000		*P*=0.151	*P*=10.000	*P*=0.000	*P*=0.000^*∗*^	*P*=0.000	*P*=0.000	*P*=0.104	*P*=0.000
Chlorsulfuron 75% WG	68.8667^*∗*^	16.6567		13.3333	71.1000^*∗*^	62.2333^*∗*^	52.2000^*∗*^	700.0000^*∗*^	34.4667^*∗*^	1000.0000^*∗*^
	*P*=0.000	*P*=0.151		*P*=0.389	*P*=0.000	*P*=0.000	*P*=0.000	*P*=0.000	*P*=0.000	*P*=0.000
Diazinon 60% EC	55.533^*∗*^	3.3233	−13.3333		57.7667^*∗*^	48.9000^*∗*^	38.8667^*∗*^	56.6667^*∗*^	21.1333^*∗*^	86.6667^*∗*^
	*P*=0.000	*P*=10.000	*P*=0.389		*P*=0.000	*P*=0.000	*P*=0.000	*P*=0.000	*P*=0.032	*P*=0.000
Glycel + agro-2,4-D	−2.2333	−54.4433^*∗*^	−71.1000^*∗*^	−57.7667^*∗*^		−8.8667	−18.9000	−1.1000	−36.6333^*∗*^	28.9000^*∗*^
	*P*=10.00	*P*=0.000	*P*=0.000	*P*=0.000		*P*=0.843	*P*=0.071	*P*=10.000	*P*=0.000	*P*=0.002
Glycel 41% EC	6.6333	−45.5767^*∗*^	−62.2333^*∗*^	−48.9000^*∗*^	8.8667		−10.0333	7.7667	−27.7667^*∗*^	37.7667^*∗*^
	*P*=0.967	*P*=0.000	*P*=0.000	*P*=0.000	*P*=0.843		*P*=0.736	*P*=0.919	*P*=0.002	*P*=0.000
Malathion 50% EC	16.6667	−35.5433^*∗*^	−52.2000^*∗*^	−38.8667^*∗*^	18.9000	10.0333		17.8000	−17.7333	47.8000^*∗*^
	*P*=0.151	*P*=0.000	*P*=0.000	*P*=0.000	*P*=0.071	*P*=0.736		*P*=0.104	*P*=0.106	*P*=0.000
Mancozeb 80% WP	−1.1333	−53.3433^*∗*^	0–700	−56.6667^*∗*^	1.1000	−7.7667	−17.8000		−35.5333^*∗*^	300.0000^*∗*^
	*P*=10.000	*P*=0.000	*P*=0.000	*P*=0.000	*P*=10.00	*P*=0.919	*P*=0.104		*P*=0.000	*P*=0.001
Endosulfan 35% EC	34.4000^*∗*^	−17.8100	−34.4667^*∗*^	−21.1333^*∗*^	36.6333^*∗*^	27.7667^*∗*^	17.7333	35.5333^*∗*^		65.5333^*∗*^
	*P*=0.000	*P*=0.104	*P*=0.000	*P*=0.032	*P*=0.000	*P*=0.002	*P*=0.106	*P*=0.000		*P*=0.000
Contol (water)	−31.133	−83.3433^*∗*^	−1000.0^*∗*^	−86.6667^*∗*^	−28.900^*∗*^	−37.767^*∗*^	−47.8000^*∗*^	−300.00	−65.5333^*∗*^	
	*P*=0.001^*∗*^	*P*=0.000	*P*=0.000	*P*=0.000	*P*=0.002	*P*=0.000	*P*=0.000	*P*=0.001	*P*=0.000	

**Table 6:** LD_50_ of pesticides tested in the laboratory and their categories.

Pesticides	LD_50_	Toxicity classification	Dose tested	^#^StandardLD_50_
Diazinon 60% EC	<0.1 *μ*l/bee	Highly toxic	0.3 *μ*l, 0.2 *μ*l, 0.1 *μ*l	Acute LD_50_ < 2 *μ*g/bee
Endosulfan 35% EC	<0.1 *μ*l/bee	Highly toxic	0.3 *μ*l, 0.2 *μ*l, 0.1 *μ*l	Acute LD_50_ < 2 *μ*g/bee
Malathion 50% EC	<0.1 *μ*l/bee	Highly toxic	0.3 *μ*l, 0.2 *μ*l, 0.1 *μ*l	Acute LD_50_ < 2 *μ*g/bee
Chlorsulfuron 75% WG	<0.1 *μ*l/bee	Highly toxic	0.3 *μ*l, 0.2 *μ*l, 0.1 *μ*l	Acute LD_50_ < 2 *μ*g/bee
2,4-D	6–8 *μ*l/bee	Moderately toxic	8 *μ*l, 6 *μ*l, 4 *μ*l	Acute LD_50_ 2–10.99 *μ*g/bee
Glycel 41% EC	44 *μ*l/bee	Slightly toxic	44 *μ*l, 33 *μ*l, 22 *μ*l	Acute LD_50_ 11–100 *μ*g/bee
Mancozeb 80% WP	33–44 *μ*l/bee	Slightly toxic	44 *μ*l, 33 *μ*l, 22 *μ*l	Acute LD_50_ 11–100 *μ*g/bee
Glycel 41% EC + 2,4-D	5–7 *μ*l/bee	Moderately toxic	9 *μ*l, 7 *μ*l, 5 *μ*l	Acute LD_50_ 2–10.99 *μ*g/bee
Dimethoate 40% EC	<0.1 *μ*l/bee	Highly toxic	0.3 *μ*l, 0.2 *μ*l, 0.1 *μ*l	Acute LD_50_ < 2 *μ*g/bee

## Data Availability

All data used to support the findings of this study are available upon reasonable request from the corresponding author.
